# Innovative teaching in infection prevention and control and infectious diseases education: testing and investigation of student perceptions

**DOI:** 10.1007/s15010-024-02332-8

**Published:** 2024-07-09

**Authors:** Hani E. J. Kaba, Martin Misailovski, Jasmin Brähler, Josué A. Bucio Garcia, Tanja Artelt, Tobias Raupach, Simone Scheithauer

**Affiliations:** 1 Department of Infection Control and Infectious Diseases (IK&I), University Medical Center Göttingen, Georg-August University of Göttingen, Göttingen, Germany; 2 Institute of Medical Education, Medical Faculty, University of Bonn, Bonn, Germany

**Keywords:** Innovative teaching methods, Infectious diseases, Infection prevention and control

## Abstract

**Purpose:**

Many curricula promote frontal teaching approaches, potentially decreasing interaction and motivation – also within infection prevention & control and infectious diseases (IPC/ID). We aimed to investigate the implementation of three innovative teaching methods (ITM) within IPC/ID education: game-based learning (GBL), peer-teaching (PT) and misinformation detection (MID).

**Methods:**

Multi-phase study involving third-year medical students was conducted. Phase-1 included a cross-sectional survey, assessing previous ITM-experience and interest to participate in phase-2, where the students were divided into teams. Each team prepared a video covering an IPC/ID-topic with deliberately placed misinformation, which had to be identified and corrected by the opposing team, followed by qualitative evaluation (phase-3). Finally, the MID-concept was incorporated into regular curricula in a non-competitive environment (phase-4) and evaluated within a cohort not involved in phases 1–3.

**Results:**

276 students responded to phase-1. 58% expressed interest in participating in phase-2. Roughly 59% [47–71%] CI-95% of respondents without previous PT-experience stated interest in PT, while the interest in GBL and MID was even higher. 19 students participated in phase-2. All topic MID-scores ranged between 6 and 8/10 points, except for emporiatrics (3/10). Post-hoc analysis revealed a positive student-perception of ITM, particularly GBL. Phase-4 received 103 responses with general positive evaluation. Major agreements existed on the usefulness of critical information evaluation for medical practice (82% [75–91%] CI-95%) and of MID during studies (69% [59–79%] CI-95%).

**Conclusion:**

our results hint at a relatively high interest in ITM and show MID applicability in regular IPC/ID curricula, which could be of advantage for the learning environment.

**Supplementary Information:**

The online version contains supplementary material available at 10.1007/s15010-024-02332-8.

## Introduction

Competency in infection prevention & control and infectious diseases (IPC/ID) is pivotal for medical practice, independent of the discipline. Yet, the cross-sectional character of IPC/ID, rather than being a core subject, leads to reduced curricular exposure of undergraduate students to these subjects, potentially hampering perception and proper IPC/ID-education and therefore motivation. The use of new forms of teaching could create a more interactive classroom environment, which could increase interest and motivation and thus ultimately competencies in order to decrease pathogen transmissions and healthcare associated infections, increasing patient safety.

There is a plethora of innovative teaching methods, especially in medical education. Game-based learning (GBL) basically refers to “learning that is facilitated by the use of a game” [1]. It is an emerging method in adult education, which has also found acceptance in medical education [2]. The benefits of peer teaching (PT) have been known for some time, both on the side of the “students” as well as on the side of the “teachers” [3]. An early and active practice of searching for (systematic) errors could help with individual actions to anticipate possible sources of error in later work [4]. The detection of system errors was additionally regarded among 13 Core Entrustable Professional Activities (EPAs), expected to be commanded by medical graduates without supervision [5].

We implemented a novel approach of teaching IPC/ID among clinical phase medicine students (undergrads), combining three innovative teaching methods (ITM) at once, within a digital setting: GBL, PT and misinformation detection (MID). We thereby investigated the attitude of future medical professionals towards these methods within IPC/ID, their general perception of IPC/ID, previous experience with the used ITM, opinions on further improving this approach as well as their implementation in the regular curriculum.

## Methods

**Fig. 1:**
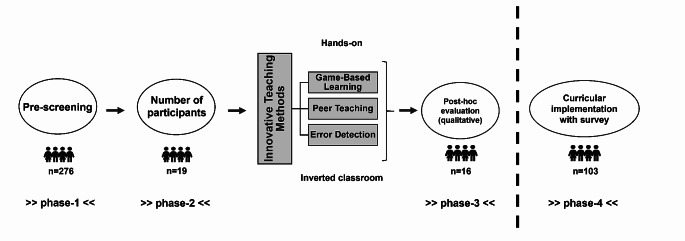
Different phases of the study

This project and the evaluation were multi-staged (Fig. 1). A monocentric, cross-sectional, anonymous survey of medical students (third year onwards) was conducted between April and May 2021 (stage 1) at the Medical Faculty of the University of Göttingen (UMG). The aim was to identify students’ preferences for additional training, perceptions towards ITM and to assess their willingness to participate in stage-2. We provided definitions for each ITM in the survey questionnaire and sent out three reminders on a weekly basis. Assessment of topics in which additional training was necessary according to students, was conducted among those who had undergone the local IPC/ID course (self-report). The survey was with voluntary participation and conducted using the open-source survey tool “LimeSurvey” (LimeSurvey GmbH, Hamburg, Germany; http://www.limesurvey.org). It was distributed electronically via E-mail by the dean’s office. Providing answers to every item of the questionnaire was voluntary. Therefore, different sample sizes could be obtained for different items.

Stage-2 included the ITM implementation. After an introductory meeting, the participants were divided into two teams. Each team was entitled to prepare a short teaching video under supervision provided by resident physicians, specialists in IPC/ID and/or life science/ public health professionals. Allocation of the video topics to the groups was random. The videos (max. length of 10 min.) covered a given IPC/ID topic in which misinformation were deliberately placed (*n* = 5). After watching the video, each opposing team had to identify and correct the misinformation within a given time frame, with all possible aids being permitted. Each identification and/or correction event was documented. Together, correct identifications and corrections made up the MID score per team. In the next step, subjective individual participant opinion on the approach in a pre-/post interventional manner was collected and qualitatively evaluated (free-form text items; stage-3), via an anonymous and voluntary questionnaire (LimeSurvey).

Finally, the concept was rolled out within the regular curriculum (winter semester 2023/24, two distinct cohorts), targeting a bigger sample of participants who have not been part of the pre-screening in stage-1. This rollout was accompanied by necessary modifications to the concept. Since the MID component was the novel element within our ITM system, we withdrew both the GBL and PT components allowing us to investigate specific student perception of MID within the same digital setting (stage-4). The study team prepared a teaching video covering “hospital outbreak management” and containing a random number of erroneous information. The video was presented to cohorts of third year students in a form of seminar, in same week when the official outbreak management lecture was given, which covered the same content. The students were given the task of critically evaluating all information provided in the video, in an open discussion (among each other and with the lecturers). Upon the end, all students were invited to voluntarily and anonymously provide their opinion on the whole session, their general perception of MID and its importance for future careers, using comparative, closed-ended (likert scale) and open-ended (free text fields) question items respectively (LimeSurvey; questionnaire link provided onsite via QR-code).

Participation at every stage was voluntary. Non-monetary incentives were awarded to the participants in stage-2, otherwise, no incentives were given.

## Results and discussion

**Fig. 2:**
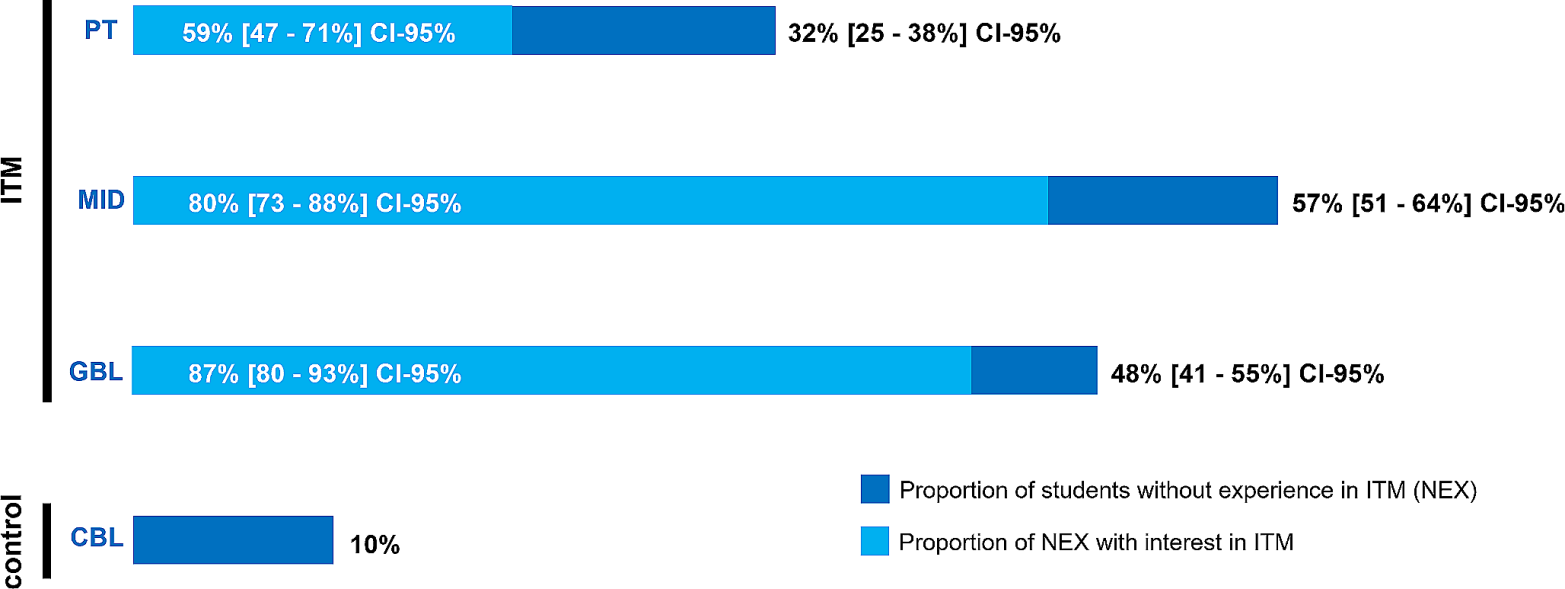
Student’s (*n* = 206) previous experience with the innovative teaching methods (ITM) covered by this work (peer-teaching, PT; game-based learning, GBL; misinformation identification; MID). Case-based learning (CBL), already established in the curriculum, was used to control proper understanding of the survey items. The dark blue bars correspond to the proportion of undergrads without experience (NEX) in the given ITM. The light blue bars correspond to the proportion of NEX with interest to experience the given ITM

276/1479 undergrads (19%) participated in stage-1 (mean age 25 ± 3 years; 70% female; semester affiliation provided in the Online Resource, Table S1). In total, 127/219 (58%, [51–61%] CI-95%) of the respondents expressed readiness for participation in stage-2. MID was less familiar for respondents than other ITM. Roughly 60% of respondents without PT-experience stated interest in this method, compared to > 80% for other ITM (Fig. 2). Interestingly, among students who have undergone the local IPC/ID course (*n* = 175/175, 100%), antimicrobial-resistance (AMR) topped the topics in which additional training was required, in contrast to “hand/ basic hygiene (HHYG)”, for which the demand was around 3%. A similar observation was made for the topic “sterilization/disinfection procedures” (10% selections, Table 1). This might reflect some overconfidence in their own basic IPC/ID skills, a known phenomenon among healthcare professionals which potentially leads to a decreased motivation of practicing basic IPC/ID measures [6].

**Table 1:** Student-reported IPC/ID topics requiring additional training (*n* = 175)

Topics	Proportion	CI-95%
Hand hygiene practice	3%	[0–5%]
Antimicrobial resistance	65%	[58–72%]
Surveillance	37%	[30–44%]
Respiratory diseases	29%	[22–36%]
Outbreak management	49%	[42–57%]
Transmission routes	20%	[14–26%]
Personal protective equipment	22%	[16–28%]
Bloodborne infections	27%	[20–33%]
Water hygiene and safety	23%	[17–33%]
Sterilization/ desinfection	10%	[6–15%]
Molecular investigation in transmission dynamics	35%	[28–42%]
Intravenous (iv) device management/ central-line associated infection prevention	47%	[40–55%]
Surgical hand desinfection	14%	[9–19%]

While almost all responding undergrads judged IPC/ID as important or very important for future professional careers (98% [96–100%] CI-95%), ca. 38% evaluated its importance for passing exams during studies (38% [31–44%] CI-95%). This might exacerbate the problem of motivation barriers even more and might result in increased risk poor hygiene practice at later career stages. Additionally, it could indicate a comparably lower influence of IPC/ID in exam passing odds.

Nineteen students participated in stage-2 (three cohorts during the summer semester 2021, one online, two onsite sessions). Team sizes varied between two and four students per team. The topics covered by the students varied for each cohort and covered AMR, emporiatrics, vancomycin utilization, healthcare-associated infections (with focus on bloodborne infections, central line-associated bloodstream-infections and IPC measures). These topics have been selected on the basis of the results obtained in stage-1. All topic MID-scores ranged between 6 and 8/10 points, except for emporiatrics (3/10).

Qualitative analysis (stage-3) of 16 entries showed three main patterns. First, the implemented ITM were largely positively evaluated, particularly observed for the GBL component, largely due to the “fun” aspect, that helps to create a good learning environment, according to the respondents. Second, while the MID component was judged to increase attention and analytical skills, concerns were raised over whether the wrong information could be accidentally memorized instead of correct information. Third, the PT component was judged with mixed feelings. The students believed that PT might create a better teaching environment due to the reduced barriers among the peers. However, success was thought to be very much dependent on the capabilities and knowledge/ skills of the peers, which mostly do not possess advanced knowledge and experience compared to full lecturers. The self-preparation of videos was largely regarded as tedious although believed to support self-education.

We obtained 103/175 responses (59%), in the rollout phase survey (stage-4). The whole MID-session was largely positively evaluated, with 66% [55–73%] CI-95% indicating that their expectations of the session have been met. Furthermore, a consensus existed on the usefulness of critical information evaluation for future professional practice (82% [75–91%] CI-95% selected “likely” or “very likely” useful, *n* = 87). Interestingly, MID was largely regarded as useful for learning during medical studies (69% [59–79%] CI-95% selected “likely” or “very likely” useful, *n* = 87). Main reasons for this positive perception were that “MID allows information to be better retained in memory” (67% selections), “correcting misinformation improves analytical skills” (60%) and/or “correcting misinformation is an innovative learning method” (40%). On the other hand, a proportion of students were critical of MID (13% [6–20%] CI-95% selected “unlikely” or “very unlikely” useful for learning during medical studies). The majority of this proportion (82%, *n* = 11) related the decision to concerns about whether erroneous information being rather memorized instead of the correct, in line with responses received in stage-3. This would indicate that such perception towards MID exist among students independent of the gaming and the hands-on (peer-teaching) elements. Furthermore, it would indicate that this same opinion exists among those who participated voluntarily in a non-curricular teaching session, as well as those for whom the participation in the session was compulsory as per curriculum. The subjective difficulty level of error identification in the displayed video (Online Resource, Table S2) showed no unequivocal association pattern with the perception of MID usefulness in education (OR = 0.70 [0.24–2.06] CI-95%).

In total, our approach complements previous efforts to incorporate GBL-approaches along MID in IPC/ID education directed towards residents, lead physicians or students [7–11]. Employing serious games has previously succeeded in increasing nursing students’ IPC/ID knowledge [11]. Beside the standardized content, the lower inhibition threshold provides an important learning advantage [10], something that was also found in the undergrads reflections on our approach. Yet, we believe that this is one of the first studies to focus on combining multiple ITMs, with an emphasis on MID, while studying the effect of single ITMs is possible through easy modification of the design, within the same background setting. Furthermore, our concept is easily adaptable to practically any topic (even outside IPC/ID) and promotes active learning and targets future medical doctors at an early stage. On the other hand, our observations have distinct limitations which restrict the extension of the surveys beyond the sample of respondents. The major limitation is that the investigation was made at a one local university (single centre). Thus, student perceptions are dependent on the previous curricular exposition and cannot be generalized. The perception of participating in stage-2 might be somehow biased due to having online and onsite participants in the same pot, due to the pandemic situation dependent restrictions. However, this could still be of advantage, allowing to cover a broader amount of information and to test different scenario settings for conducting such teaching projects.

We believe that our approach fills a still existing gap in IPC/ID-education research and provides an excellent basis for further improvement and testing in larger, probably multi-centric settings. This could potentially be achieved by further analysing the influence of MID, in a non-competitive environment, on student related outcomes (subjective knowledge gain, MID hits, satisfaction or eventually exams grades), thus allowing a matrix of combinations in order to find the best possible teaching format. A new research grant for this purpose has already been obtained.

## Electronic supplementary material

Below is the link to the electronic supplementary material.

Supplementary Material 1

## Data Availability

Supplementary data is present in the Online Resource (suppl. information file).
