# Supplementary material for: Innovative teaching in infection prevention and control and infectious diseases education: testing and investigation of student perceptions
Source: Infection. 2024 Jul 9;53(1):139–43. doi: 10.1007/s15010-024-02332-8 (PMC11825523; doi:10.1007/s15010-024-02332-8)
Supplement: Supplementary file 1 — Supplementary Material 1 [file 15010_2024_2332_MOESM1_ESM.docx]

**Supplemental information**

Infection

**Innovative teaching in infection prevention and control and infectious diseases education: Testing and investigation of Student Perceptions**

Hani E. J. Kaba^1^*, Martin Misailovski^1^* ^a^, Jasmin Brähler^1^*, Josué A. Bucio Garcia^1^, Tanja Artelt^1^, Tobias Raupach^2^, Simone Scheithauer^1^

^1^Department of Infection Control and Infectious Diseases (IK&I), University Medical Center Göttingen, Georg-August University of Göttingen, Germany

^2^Institute of Medical Education, Medical Faculty, University of Bonn, Germany

^a^corresponding author

*shared first authorship

Corresponding Author:

Prof. Dr. med. Simone Scheithauer

E-Mail-Adress: krankenhaushygiene.leitung@med.uni-goettingen.de

Suppl. Table S1. Distribution of responding students according to semester affiliation. Please note that 37 respondents (of total n = 276) did not provide information of semester affiliation.

| Clinical semester | n | proportion | CI-95% |
| --- | --- | --- | --- |
| 1st | 36 | 15% | [11 – 20%] |
| 2nd | 30 | 13% | [8 – 17%] |
| 3rd | 38 | 16% | [11 – 21%] |
| 4th | 41 | 17% | [12 – 22%] |
| 5th | 32 | 13% | [9 – 18%] |
| 6th | 20 | 8% | [5 – 12%] |
| internship year | 42 | 18% | [13 – 22%] |
| sum | 239 |  |  |

Suppl. Table S2. Distribution of responses in phase 4, towards evaluating the difficultness of error detection in the roll-out video (n = 103).

| Selection | frequency | proportion | CI-95% |
| --- | --- | --- | --- |
| very easy | 2 | 2% | [-1 – 5%] |
| easy | 25 | 24% | [16 – 33%] |
| neither easy nor difficult | 48 | 47% | [37 – 56%] |
| difficult | 20 | 19% | [12 – 27%] |
| very difficult | 3 | 3% | [0 – 6%] |

no selection: n = 5
